# Harnessing the Molecular Fingerprints of B Cell Lymphoma for Precision Therapy

**DOI:** 10.3390/jcm11195834

**Published:** 2022-10-01

**Authors:** Afua Adjeiwaa Mensah, Patrizia Mondello

**Affiliations:** 1 Institute of Oncology Research, Faculty of Biomedical Sciences, USI, 6500 Bellinzona, Switzerland; 2 Division of Hematology, Mayo Clinic, Rochester, MN 55905, USA

The last two decades have brought ground-breaking advances in genetics, culminating in deep profiling of the human genome and high resolution detection of genetic variants. These developments have rewired preclinical and clinical efforts in understanding cancer, paving the way for precision therapies [1]. Lymphomas are among the most frequent tumours worldwide, with B cell lymphomas accounting for approximately 85% of newly diagnosed cases [2]. They arise from the germinal centre (GC), a specialised structure within the B cell follicle that forms upon encounter of B cells with an external antigen. Similarly to other cancers, the ability of lymphoma cells to bypass physiological signalling pathways controlling proliferation, differentiation and cell death is primarily governed by genetic and epigenetic defects. In line with this, the most recent classifications of lymphoma subtypes largely rely on the presence or absence of specific mutated genes and concordant aberrant molecular signatures [3,4]. As we gain more insights into the genetic evolution of lymphomas, either as they progress towards a more aggressive disease or because of resistance mechanisms arising in response to treatment, it is becoming increasingly evident that therapies targeting lymphoma-specific proteins represent important components of the therapeutic arsenal [5,6]. Personalised treatments, also known as targeted or precision medicines, utilise the molecular fingerprint of a patient’s tumour to inform drug development and treatment choices [7]. Due to the increased antitumour specificity of targeted agents compared to their non-targeted counterparts, precision medicine is currently the focus of most efforts to develop anti-lymphoma drugs. The turning point in the development of effective targeted therapies for lymphoma came with our increased understanding of the molecular drivers of this disease. The massive amounts of data gleaned from high-throughput sequencing of samples from multiple patient cohorts have provided a molecular basis for the observed clinical differences in lymphoma progression, response to therapy, and survival in individual lymphoma patients, and have provided a rationale for the development of therapies targeting specific lymphoma drivers [3,4]. 

The first targeted agent approved for treating B cell lymphoma was the monoclonal antibody rituximab (R), which has significantly improved patient survival in combination with chemotherapy (R-CHOP), compared to chemotherapy alone [8]. Following on from rituximab, multiple monoclonal CD20 antibodies have been developed and approved for lymphoma treatment including ofatumumab and obinutuzumab, but none have shown superiority to R-CHOP [9,10,11,12]. More recently, bispecific T cell engager (BiTE) antibodies that bind a B-cell-specific antigen on tumour cells (usually CD19) and CD3 on T cells have shown promise. The first-in-class BiTE, blinatumomab, was first approved for B cell acute lymphoblastic leukaemia [13], but its use is now expanding to other B cell lymphomas in both relapsed and frontline settings [14,15]. By bringing tumour cells in proximity to T cells, BiTEs enhance tumour cell killing by direct cell-to-cell contact. BiTEs offer an off-the-shelf and more immediate alternative to cellular therapy with chimeric antigen receptor T-cells (CAR-T). The latter require weeks to engineer autologous T cells expressing a chimeric T cell receptor that targets a cell surface antigen such as CD19. Additionally, BiTEs show lower grade, albeit similar treatment-related toxicities, to CAR-T cells. The mechanism of action of a BiTE can also be its Achille’s heel—the reliance on the patient’s T cells to kill tumour cells makes BiTEs unsuitable when T cell activity is poor. While it might appear that BiTEs and CAR-T cells compete for the same clinical field, recent data suggest the two can improve patient outcome when used sequentially, with CD20 BiTEs showing efficacy after failure of CD19 CAR-T cell therapy [16]. Resistance mechanisms to both platforms are T cell exhaustion secondary to repeated and/or prolonged antigen exposure and increased expression of checkpoint inhibitor molecules such as PD-L1 [17,18].

Monoclonal antibodies conjugated to cytotoxic payloads via a chemical linker, so-called antibody-drug conjugates (ADCs), represent a promising advancement on monoclonal antibodies, since they combine the tumour-homing function of a monoclonal antibody with a highly cytotoxic payload [19]. As ADCs are internalized after antibody recognition of a tumour-specific antigen followed by payload release inside the cell, the toxins conjugated to the antibody moiety of ADCs typically target intracellular macromolecules including tubulin, DNA and RNA polymerase II. The CD79 ADC polatuzumab-vedotin combined with rituximab alone or with both rituximab and bendamustine has shown efficacy in relapsed/refractory (R/R) diffuse large B cell lymphoma (DLBCL). Notably, polatuzumab-vedotin confers a survival advantage when used as a replacement for vincristine within the frontline R-CHOP regimen [20,21,22]. 

The development of novel therapeutic agents aimed at disrupting well-defined oncogenic signalling pathways has also been extensively explored in the last decade. Small molecules targeting kinases have shown robust pre-clinical and clinical activity in lymphomas, leading to the approval of three Bruton’s tyrosine kinase (BTK) inhibitors and three phosphatidylinositol 3-kinase (PI3K) inhibitors [23,24,25,26,27,28]. The efficacy of these molecules in lymphoma treatment highlights the major role of aberrant signalling cascades in driving and/or sustaining lymphomagenesis. The dynamic nature of these pathways and their convergence on each other might explain the development of resistance mechanisms when one of these components is targeted [29]. This equally suggests the potential for novel combinations or sequential approaches that block putative feedback mechanisms and treatment escape routes. However, while these rational combinations enhance tumour cytotoxicity, they also increase treatment associated toxicities and adverse events. In line with this notion, a plethora of clinical trials have tried adding targeted therapies to frontline R-CHOP regimen in an attempt to enhance efficacy but none have succeeded in improving survival [7]. This was partly due to the additional toxicities associated with these novel agents that impaired the achievement of the target dose, thus limiting overall efficacy.

Another recently developed treatment strategy involves targeting adaptive immune escape mechanisms to unmask cancer cells and in turn reactivate immune surveillance. Monoclonal antibodies targeting PD-L1, PD-1 and CTLA-4 are the big players in this approach, but while they have been game changers in many solid tumours, lymphomas have failed to show robust responses. The factors governing the lack of response, as well as early progression in lymphoma patients treated with immune checkpoint therapy, remain obscure, but might be related to an unfavourable tumour microenvironment (TME) and/or genetic alterations that favour immune escape. Notorious exceptions to this are Hodgkin’s lymphoma and primary mediastinal B cell lymphoma [30,31], likely due to the robust expression of PD-L1 in these diseases. Among lymphomas unresponsive to immune checkpoint therapy, DLBCL and follicular lymphoma frequently present genetic alterations conducive to immune escape that target transcription factors and epigenetic modifiers with roles in shaping the lymphoma TME [32]. These include mutations in CREBBP and EZH2 that lead to loss of MHC-I and MHC-II, while selective inhibition of their counterbalance proteins, e.g., HDAC3, or of the mutated proteins themselves, e.g., EZH2, restores antigen presentation and, in turn, an anti-lymphoma immune response [33,34,35]. The correct functioning of epigenetic proteins can also be influenced by the availability of metabolic products such as S-adenosyl methionine and acetyl-CoA, which are essential donors for epigenetic methylation and acetylation reactions, respectively, thereby intricately linking epigenetic regulation and metabolism with the lymphoma TME. Since metabolic subtypes of DLBCL show differential responses to epigenetic agents [36], the combination of specific metabolic inhibitors with other targeted agents such as selective histone deacetylase inhibitors could be therapeutically beneficial. 

The classes of targeted agents presented here, and their mechanisms of action, demonstrate that we are on the cusp of a new molecular era, whose success will require a deeper understanding of the processes underlying lymphomagenesis. Much more work needs to be done to identify predictive biomarkers and develop simpler platforms for the routine identification of molecular subgroups. Our improved understanding of these mechanisms will inform the design of more effective therapeutics with less toxicity, which target synthetic vulnerabilities while promoting antitumour immunity.

We have just begun to decipher the molecular fingerprints of B cell lymphomas and, as such, routine personalised medicine is still a long way off. However, the strides made thus far are encouraging and fuel an expectation for the future implementation of personalised treatments in standard clinical practice.
